# Autonomic etiology of heart block in amyotrophic lateral sclerosis: a case report

**DOI:** 10.1186/1752-1947-8-224

**Published:** 2014-06-24

**Authors:** Kamal Shemisa, David Kaelber, Sahil A Parikh, Judith A Mackall

**Affiliations:** 1 University Hospitals Case Medical Center, Cleveland, OH, USA; 2 Metrohealth Systems Cleveland, Ohio, USA; 3 University Hospitals Department of Internal Medicine, 11100 Euclid Avenue, Cleveland, OH 44106, USA

**Keywords:** ALS, Arrhythmia, Autonomic dysfunction, Denervation, Heart block

## Abstract

**Introduction:**

The cardiovascular consequences related to amyotrophic lateral sclerosis are relatively underappreciated. The disease invokes a systematic degeneration of autonomic neurons leading to autonomic dysfunction. We therefore hypothesized that patients with amyotrophic lateral sclerosis may have a predilection to the development of cardiac conduction disorders.

**Case presentation:**

A 65-year-old Caucasian man with advanced amyotrophic lateral sclerosis presented with progressive dyspnea and palpitations. A previous evaluation attributed his dyspnea to neuromuscular weakness and he underwent a pulmonary evaluation. Pulmonary function tests did not indicate a worsening from baseline. An electrocardiogram was obtained which demonstrated new third degree atrioventricular block. A previously obtained electrocardiogram indicated normal sinus rhythm. On echocardiogram, a structurally normal heart was observed without significant valvular disease. He was offered a permanent dual chamber pacemaker for definitive treatment, however, he declined.

**Conclusions:**

We believe that his symptoms were probably attributable to atrioventricular block. Patients with advanced amyotrophic lateral sclerosis experience loss of heart rate variability and enhanced vasomotor responses. As patients progress later in the disease, sympathetic denervation and vagal predominance contribute to the development of atrioventricular block. We conducted a query using the Explorys database of patients with amyotrophic lateral sclerosis and heart block. The prevalence of heart block was estimated to be 25% higher in patients with the disease as compared to the general population. This is the first reported case that attempts to describe the relationship of atrioventricular block with amyotrophic lateral sclerosis.

## Introduction

The cardiovascular consequences related to amyotrophic lateral sclerosis (ALS) are relatively underappreciated. Well-characterized consequences of the illness are derangements related to the autonomic nervous system which include critical mechanisms involved in regulating blood pressure, heart rate, and other homeostatic functions such as digestion and temperature regulation. ALS affects the autonomic nervous system initially by invoking degeneration of neurons that lead to sympathetic predominance and vagal withdrawal. In ALS there are losses of the baroreflex response and perturbations of preganglionic sympathetic ganglia. We therefore hypothesized that patients with ALS may have a predilection to the development of cardiac conduction disorders. We then examined the prevalence of heart block via a national database and conducted a literature search to explain the pathogenesis of heart block in a patient with ALS.

## Case presentation

**Figure 1:**
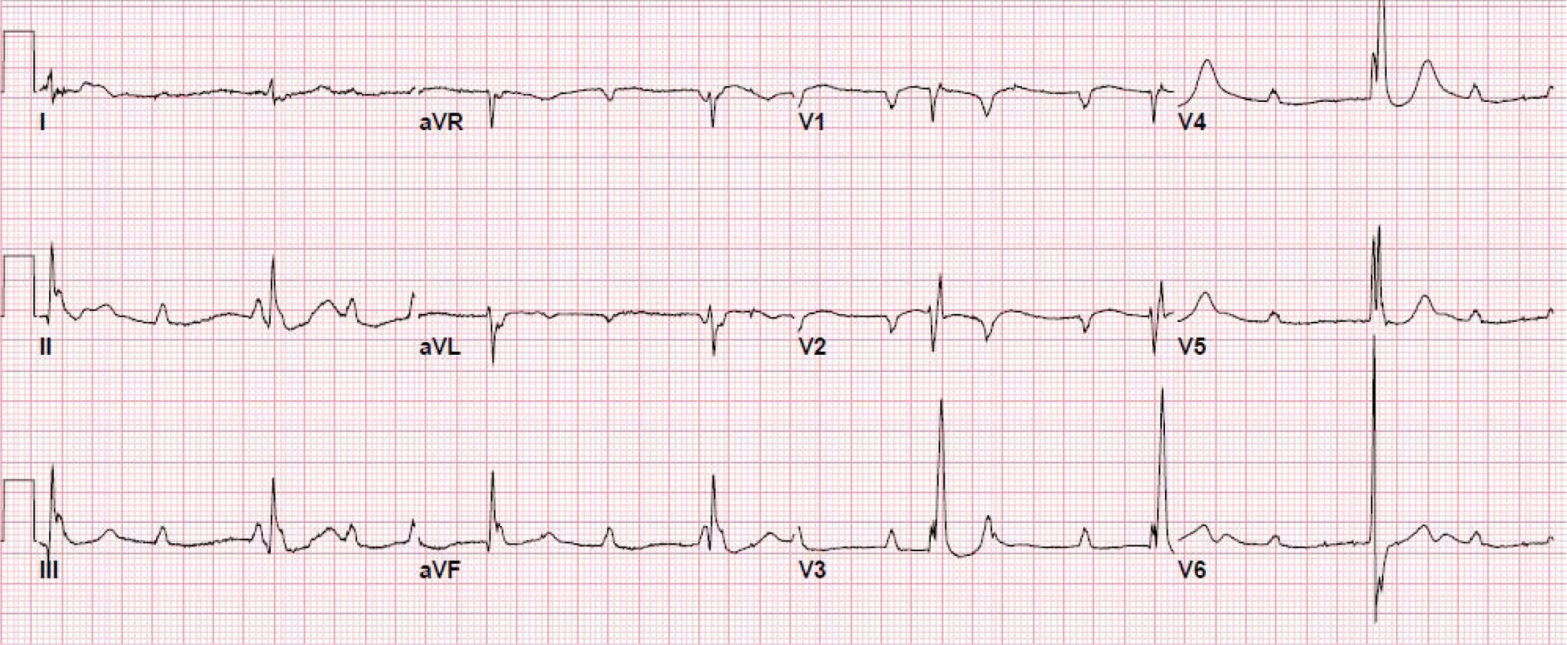
On this 12-lead electrocardiogram, the patient has complete heart block with an escape rhythm of 50 beats per minute.

Our patient was a 65-year-old Caucasian man with an 18-month history of ALS, who presents with progressive dyspnea and palpitations. A previous evaluation of his dyspnea attributed the cause to neuromuscular weakness and he underwent a pulmonary evaluation. A chest X-ray demonstrated clear lung fields. His arterial blood gas revealed a pH 7.4, carbon dioxide partial pressure 62, oxygen partial pressure 117, on fraction of inspired oxygen 40% and concomitant blood chemistry demonstrated elevated serum bicarbonate, both consistent with chronic carbon dioxide retention. Pulmonary function testing indicated a forced expiratory volume in 1 second (FEV1) of 79% and FEV1/forced vital capacity of >80%. These pulmonary function tests did not represent a significant decrement from baseline.An electrocardiogram (ECG ) was obtained which demonstrated hemodynamically stable new third degree atrioventricular (AV) block with a junctional escape rate of 50 beats per minute (Figure 1). A previously obtained ECG indicated normal sinus rhythm. On further review, he was not prescribed medications with AV nodal blocking properties while at home or during his hospitalization. On echocardiogram, a structurally normal heart was observed with normal left ventricular dimensions and function without significant valvular disease. He was offered a permanent dual chamber pacemaker for definitive treatment, however, he declined.

We believe that his symptoms were probably attributable to his AV block. The original symptoms of dyspnea with palpitations were initially attributed to ALS neuromuscular weakness during periods of exertion. The narrow complex escape rhythm suggested the block occurred at or above the level of the AV node, an area innervated by the autonomic nervous system. The role of ALS as the etiology for the development of AV block has not been previously reported. However, given the association of autonomic dysfunction with ALS, conduction abnormalities might be expected.

Determining associations of disease can be difficult and often requires large data sets to generate conclusions. The Explorys platform is a system that aggregates standardized and normalized electronic health record data currently from 13 healthcare systems around the USA and thousands of providers’ systems representing 15 million patients. We conducted a query using the Explorys Explore tool of patients with ALS and any degree of heart block (1st, 2nd, and 3rd degree) using International Classification of Diseases, Ninth Revision, diagnosis codes for both conditions (ALS = 335.20, Heart block = 426.0, 426.11, 426.12, 426.13). We sought to determine the difference between the prevalence of heart block in the general population and in those with ALS.

In Explorys, there was a base of 14,092,470 patients of which 3470 had ALS. Heart block was present in 73,801 patients without ALS and in 30 patients with ALS. The prevalence in the diagnosis of heart block in the general population was 0.52%. In ALS the prevalence of the diagnosis of heart block was 0.86%. The difference in prevalence of heart block was therefore estimated to be 25% higher in patients with ALS (P-value 0.01; confidence interval 0.002 to 0.100).

## Discussion

ALS is a neurodegenerative disorder affecting both upper and lower motor neurons. Patients eventually succumb to total paralysis and respiratory failure. Research efforts have focused on the improvement and prevention of further neuromuscular deterioration and the management of respiratory failure.

Cardiovascular consequences related to ALS are relatively underappreciated. In fact, it is believed that concomitant heart diseases are rare in ALS [1]. Like Parkinson’s disease and Shy–Drager syndrome, ALS affects the autonomic nervous system which contributes to regulation of heart rate, blood pressure and other homeostatic functions. In fact, the course of autonomic dysfunction begins with vagal withdrawal and sympathetic predominance followed by sympathetic denervation and vagal predominance. Therefore, it is not surprising that patients with ALS may experience palpitations, dizziness, dyspnea and syncope [2,3]. However, there is little understanding of the contribution of ALS to cardiac arrhythmias. Given that patients with ALS frequently have predominantly respiratory symptoms, diagnosis and treatment of cardiac arrhythmias can be difficult. An appreciation that impairments in the autonomic reflex loop contribute to these cardiac manifestations and are part of the natural progression of disease is necessary for detection and diagnosis.

### Early autonomic dysfunction

Autonomic dysfunction in ALS is common. Autonomic aberrations were first reported in small series that showed reductions in blood pressure during sustained hand grip [4]. Later observations described a discrepancy between sympathetic and parasympathetic tone. In fact, patients with ALS often exhibit bulbar dysfunction impacting centrally mediated vagal and sympathetic nerves with resultant greater autonomic dysfunction.

The impact of ALS upon the autonomic nervous system is sympathetic predominance and vagal withdrawal [5,6]. Cardiovascular autonomic testing which included analysis of heart rate variability and baroreflex function analysis were used to study those effects [2,3]. The consequences of disruptions in the autonomic reflex loop on the cardiovascular system include chronotropic incompetence and vasomotor instability. Previous research measuring muscle sympathetic nerve activity indicates higher levels of sympathetic activation in ALS [2,7]. Animal studies using the ALS-mouse model (the superoxide dismutase 1, SOD1, knockout mouse) have demonstrated persistent mydriasis with infusion of morphine compared to wild type. This suggests greater sympathetic nervous activity in the ALS mouse [8,9].

In addition, in ALS there is a loss of baroreflex response and perturbations of preganglionic sympathetic ganglia [10,11]. Studies using baroreflex stimulation indicate impaired vagal responses with preservation or enhancement of sympathetic activation [5]. For example, elevated blood pressure in ALS can be described as disproportionate alpha-sympathetic hyperactivity. Further, patients with ALS experience loss of heart rate variability and enhanced vasomotor responses [3,12]. Discordance between blood pressure and heart rate reflects sympathetic and parasympathetic nervous system imbalance.

### Late autonomic dysfunction

Cardiovascular autonomic dysfunction contributes to the morbidity of the disease. Perhaps the progression of autonomic dysfunction in ALS is under-reported. As previously described, sympathetic predominance with vagal withdrawal tends to occur early on in disease. However, in advanced ALS, the spinal cord sympathetic ganglia known as the intermediolateral nucleus also undergoes degeneration [6,13,14]. In addition to late central sympathetic failure, there is also degeneration of peripherally mediated sympathetic nerves. Electrophysiological testing using sympathetic skin response demonstrated that sequential loss of sympathetic efferents occurs late in disease progression [15]. Evidence of cardiac sympathetic denervation using metaiodobenzylguanidine-single-photon emission computed tomography (SPECT) has also been shown [16]. One marker of cardiac sympathetic denervation is ECG-associated QT interval prolongation [13]. Heterogeneous QT prolongation may predispose patients to potentially life-threatening arrhythmias.

### Cardiac arrhythmias

Arrhythmias associated with ALS may occur as a result of autonomic dysfunction. The most common arrhythmic manifestations are sinus tachycardia resulting from vagal withdrawal, interval prolongations of the QT interval, and bradycardia due to sympathetic denervation [10]. Disruptions in the autonomic reflex loop prevent sympathetic activation of vasomotor alpha-receptors to interrupt tachycardia via vagal mechanisms.

In drug treatment studies, abnormal augmentation of sympathetic tone in ALS make directed treatments for hypertension and tachycardia difficult. In infusion experiments with low doses of phentolamine, an alpha-antagonist, abrupt drops in blood pressure occurred and only modest increases in heart rate were observed. Likewise beta-antagonism with propranolol leads to considerable decreases in both blood pressure and heart rate with prolonged bradycardia [17]. As reported in one study, sympathetic denervation causes delays in cardiac repolarization leading to QT prolongation [13].

Bradycardia likewise can be explained in ALS by sympathetic denervation followed by the predominance of vagal function. Autonomic dysfunction begins with vagal release with enhanced sympathetic tone causing tachycardia. As patients later progress in ALS, sympathetic denervation occurs, followed by vagal predominance, leading to the development of bradycardia [6,13]. In a study examining the effects of vagal tone on the sinoatrial node and AV node, in patients with history of syncope, sinus arrest or bradycardia, it was found that increased vagal tone contributed to prolongation in the sinus cycle length as well as the AV node. Given that the prolongation across the AV node is a vagally mediated process, the conditions for enhanced AV nodal blockade are likely exaggerated in patients with advanced ALS [18].

## Conclusions

The cardiovascular manifestations of ALS are often an understated problem encountered through the course of the disease. Concomitant autonomic dysfunction in ALS seems to play a large role in the pathogenesis and the natural history for both vasomotor instability and cardiac arrhythmias. While ALS is a progressive illness that leads to eventual respiratory failure from neuromuscular weakness, the autonomic dysfunction is similarly progressive. It begins with vagal disruption, followed by enhanced sympathetic activity and eventually sympathetic denervation with vagal predominance.

The degree of autonomic dysfunction may offer insight into the natural course of the disease. As in the patient presented, AV nodal block argues that sympathetic denervation had occurred and suggests advanced ALS. Cardiac arrhythmias are often untreated and symptoms are often attributed to a pulmonary or psychiatric cause. This is the first reported case of complete heart block occurring in ALS.

While the findings are not well documented in the literature, the findings are well supported by previous physiological studies in ALS as well as a survey from a reputable database. Utilizing the data from the Explorys platform, there was a higher prevalence of heart block in patients with ALS. While the small number of affected individuals confers some statistical uncertainty, it suggests that a degree of AV block occurs more frequently in patients with ALS. Autonomic dysfunction occurs in other neurodegenerative illnesses and may shed light into their disease progression as well as potential treatment.

## Consent

Written informed consent for publication could not be obtained despite all reasonable attempts at contacting our deceased patient's next of kin. Every effort has been made to protect the identity of our patient and there is no reason to believe that our patient would have objected to publication.

## Competing interests

The authors declare that they have no competing interests.

## Authors’ contributions

KS, SP and JM helped with the contribution to the conception and design. DK and KS conducted the data acquisition analysis and interpretation of data. All authors assisted in drafting and revising the article. All authors were critically important for the intellectual content of the article. All authors read and approved the final manuscript.
